# Antimicrobial Susceptibility of Standard Strains of Nontuberculous Mycobacteria by Microplate Alamar Blue Assay

**DOI:** 10.1371/journal.pone.0084065

**Published:** 2013-12-30

**Authors:** Guilian Li, Lu-lu Lian, Li Wan, Jingrui Zhang, Xiuqin Zhao, Yi Jiang, Li-li Zhao, Haican Liu, Kanglin Wan

**Affiliations:** 1 National Institute for Communicable Disease Control and Prevention, Chinese Center for Disease Control and Prevention and State Key Laboratory for Infectious Disease Prevention and Control, Beijing, China; 2 Collaborative Innovation Center for Diagnosis and Treatment of Infectious Diseases, Hangzhou, China; 3 Pathogenic Biology Institute, University of South China, Hengyang, Hunan, China; 4 Key Lab of Laboratory Medicine, Wenzhou Medical College, Wenzhou, Zhejiang, China; St. Petersburg Pasteur Institute, Russian Federation

## Abstract

In this study, 24 standard nontuberculous mycobacteria (NTM) species strains including 12 slowly growing mycobacteria strains and 12 rapidly growing mycobacteria strains were subjected to drug susceptibility testing using microplate Alamar Blue assay-based 7H9 broth. The most active antimicrobial agents against the 24 NTM strains were streptomycin, amikacin, the fluoroquinolones, and the tetracyclines. *Mycobacterium chelonae*, *Mycobacterium abscessus*, *Mycobacterium bolletii,* and *Mycobacterium simiae* are resistant to most antimicrobial agents. The susceptibility results of this study from 24 NTM standard strains can be referenced by clinicians before susceptibility testing for clinical isolates is performed or when conditions do not allow for susceptibility testing. The application of broth-based methods is recommended by the Clinical and Laboratory Standards Institute, and the documentation of the susceptibility patterns of standard strains of mycobacteria can improve the international standardization of susceptibility testing methods.

## Introduction

Although the prevalence of tuberculosis is decreasing globally, increased numbers of nontuberculous mycobacteria (NTM) have been reported in human infections in recent years because of the growing number of immunosuppressed patients coupled with better diagnostic techniques [1]. Classified into rapidly growing mycobacteria (RGM) and slowly growing mycobacteria (SGM), NTM are opportunistic pathogens that can cause a wide variety of disseminated or localized diseases, particularly pulmonary, skin, and soft tissue infections. Due to the differences between even individual NTM strains, these organisms require individualized treatment that must be selected on the basis of results obtained from in vitro drug susceptibility tests (DST).

With the evolution of assay techniques, especially the wide application of a new commercially available DNA strip assay (GenoType Mycobacterium, Hain Lifescience, Nehren, Germany) [2], [3], *Mycobacteria* can be easily identified to the species level; however, our knowledge about the susceptibility patterns of NTM is limited. Our results presented here about the susceptibilities of 15 antibiotic agents against standard NTM strains could be highly valuable for clinicians.

The methods for antimycobacterial susceptibility testing include the Clinical and Laboratory Standards Institute (CLSI) broth-based methodology [4], E-test [5], agar-based testing methods [6], and the disk elution and diffusion method [7], [8]. The CLSI currently recommends Mueller-Hinton broth-based methods for RGM and Mueller-Hinton or 7H9 broth-based methods supplemented with OADC or acid-albumin-dextrose-catalase (ADC) for SGM [4]. Another broth-based method, the microplate Alamar Blue assay, has been used for years with favorable results for *Mycobacterium tuberculosis* complex isolates [9], [10]. In this study, we used the microplate Alamar Blue assay to test the activities of 15 drugs against 24 standard NTM strains in China.

## Methods

### Strains

Twenty-four NTM standard strains including 12 RGM and 12 SGM were included in this study (Table 1).

**Table 1:** MIC (µg/mL) of the 15 antimicrobial agents* to the 24 standard NTM strains.

Species(Code)		RIF	INH	STR	AK	KM	CP	OF	LOF	CAP	CEF	DOX	MIN	ETH	PAS	DIP
*M. abscessus* (ATCC19977)	RGM	>256	>256	32	32	128	4	32	16	>256	64	>256	>42.5	16	>256	>256
*M. chelonae* (NCTC946)	RGM	64	>256	16	32	64	**1**	4	**2**	>256	>256	>256	>42.5	16	>256	>256
*M. fortuitum* (ATCC6841)	RGM	64	>256	128	**4**	8	**<0.25**	**<0.25**	**<0.5**	>256	32	>256	>42.5	64	>256	>256
*M. peregrinum* (ATCC14467)	RGM	2	>256	32	**<0.5**	4	**<0.25**	**<0.25**	**<0.5**	>256	**8**	64	**10.7**	**4**	>256	>256
*M. doricum* (ATCCBAA565)	RGM	64	>256	64	**4**	16	**<0.25**	**0.5**	**<0.5**	>256	>256	>256	>42.5	32	>256	>256
*M. obuense* (ATCC27023)	RGM	16	>256	**<0.5**	**1**	4	**<0.25**	**0.5**	**<0.5**	>256	>256	2	**1.3**	8	>256	>256
*M. phlei* (ATCC11758)	RGM	**<0.5**	>256	**<0.5**	**<0.5**	8	**0.5**	**1**	**<0.5**	>256	**16**	**<0.5**	**3**	**4**	>256	>256
*M. duvalii* (ATCC43910)	RGM	**<0.5**	>256	**<0.5**	**<0.5**	**1**	**<0.25**	**<0.25**	**<0.5**	>256	**8**	**<0.5**	**0.3**	**2**	>256	>256
*M. parafortuitum* (ATCC19686)	RGM	4	>256	**<0.5**	**1**	4	**<0.25**	**<0.25**	**<0.5**	>256	>256	**<0.5**	**0.3**	8	>256	>256
*M. gilvum* (ATCC43909)	RGM	**<0.5**	>256	**4**	**1**	**2**	**<0.25**	**<0.25**	**<0.5**	>256	>256	**1**	**1.3**	**2**	>256	>256
*M. flavescens* (ATCC14474)	RGM	16	>256	**2**	**<0.5**	**1**	**<0.25**	**0.5**	**<0.5**	>256	>256	**1**	**0.7**	8	>256	>256
*M. bolletii* (CIP 108541)	RGM	>256	>256	128	**16**	64	8	64	32	>256	64	>256	>42.5	16	>256	>256
*M. intracellulare* (ATCC13950)	SGM	2	>256	**4**	**4**	16	**1**	16	4	>256	>256	4	**1**	8	>256	>256
*M. xenopi* (NCTC10042)	SGM	1	>256	**1**	**1**	-	<0.25	<0.25	<0.5	256	64	**<0.5**	**<0.08**	8	>256	>256
*M. senegalense* (ATCC35796)	SGM	2	>256	16	**2**	**2**	2	4	**1**	>256	**16**	**1**	**0.17**	8	>256	>256
*M. gordonae* (ATCC14470)	SGM	**<0.5**	>256	**1**	**2**	4	**1**	2	**0.5**	32	>256	4	**5**	**4**	>256	>256
*M. marinum* (ATCC927)	SGM	**<0.5**	>256	**4**	**1**	8	4	16	32	>256	32	**<0.5**	**0.7**	**4**	>256	128
*M. kansasii* (ATCC12478)	SGM	**<0.5**	>256	**1**	**4**	4	**<0.25**	**0.5**	**<0.5**	>256	**8**	2	**2.7**	**4**	>256	>256
*M. scrofulaceum* (ATCC19981)	SGM	**0.5**	>256	**1**	**2**	4	**<0.25**	**1**	**<0.5**	4	>256	2	**1**	**2**	>256	>256
*M. malmoense* (ATCC29571)	SGM	1	>256	**2**	**2**	4	**0.5**	**1**	**<0.5**	>256	>256	8	**5**	**4**	>256	>256
*M. avium* (ATCC25291)	SGM	8	>256	**1**	**2**	16	2	4	**2**	>256	>256	32	**11**	**4**	>256	>256
*M. szulgai* (NCTC10831)	SGM	**<0.5**	>256	**2**	**4**	8	**1**	2	**2**	>256	**16**	4	**2.7**	8	>256	>256
*M. terrae* (ATCC15755)	SGM	1	>256	**4**	**8**	16	**<0.25**	**<0.25**	**<0.5**	>256	**4**	8	**1**	16	>256	>256
*M. simiae* (ATCC25275)	SGM	8	>256	16	**8**	64	32	64	16	>256	>256	>256	>42.5	16	>256	>256

MIC, minimum inhibitory concentration; NTM, nontuberulous mycobacteria; RGM, rapidly growing mycobacteria; SGM, slowly growing mycobacteria; RIF, rifampicin; INH, isoniazid; STR, streptomycin; AK, amikacin; KM, kanamycin; CP, ciprofloxacin; OF, ofloxacin; LOF, levofloxacin; CAP, capreomycin; CEF, cefoxitin; DOX, doxycycline; MIN, minocycline; ETH, ethionamide; PAS, P-aminosalicylic acid; DIP, dipasic.

bold typeface indicates that the species was susceptible to the antimicrobial drug, while underlining indicates that the species was moderately susceptible to the antimicrobial drug.

### Antibiotics and Chemicals

Middlebrook 7H9 broth and ADC supplement were purchased from Difco (Detroit, MI, USA). Powders of 15 antimicrobial agents including rifampicin, isoniazid, streptomycin, amikacin, kanamycin, ciprofloxacin, ofloxacin, levofloxacin, capreomycin, cefoxitin, doxycycline, minocycline, ethionamide, and p-aminosalicylic acid were purchased from Sigma–Aldrich Company (St. Louis, USA), while dipasic was purchased from Chongqing Huapont Pharmaceutical Company (Chongqing, China). Alamar Blue was purchased from AbD Serotec (Oxford, UK). All of the antibiotic solutions were prepared before the day of the experiment and stored at −70°C.

### Antimicrobial Susceptibility Testing

Susceptibility testing was performed using the Middlebrook 7H9 broth microdilution method. All tests for each strain were carried out at least in duplicate. The isolates were grown on microplates. The inocula were prepared from actively growing bacteria collected from Lowenstein-Jensen slants. The strains were then adjusted with saline to a bacterial cell density of 3.0×10^8^ (McFarland 1.0 standard), and then adjusted to a 1∶20 dilution with Middlebrook 7H9 Supplement (7H9-S) (7H9 broth +10% ADC + 0.5% glycerol). Antibiotics were serially diluted twofold in 100 µL of 7H9-S. The range of antibiotic concentrations was 256–0.5 µg/mL except for ciprofloxacin and ofloxacin, which were 128–0.25 µg/mL, and minocycline, which was 42.6–0.04 µg/mL. The final reaction volume was 200 µL (100 µL of antibiotic solution and 100 µL of bacterial suspension). Three negative controls were set in this study. Drug free control well(7H9-S+inoculum) was used to decide the time of adding alamar blue. The medium(7H9-S) without inoculum control well was used to decide the interference of 7H9-S to alamar blue and a series control wells of rifampicin concentration gradients of rifampicin and 7H9-S mixture were also used to decide the interference of the color of rifampicin-7H9-S mixture to alamar blue. The plates were sealed in individual Ziploc bags and then incubated at 37°C.

After 24 h, the first drug-free growth control wells were examined using indicator (20 µL of Alamar Blue and 50 µL of sterile 5% Tween-80). The plates were then re-incubated for 8 h. If the control well turned pink, all of the other wells received the indicator. After a further 24 h of incubation, the colors of all wells were recorded. If the first drug-free growth control well did not change to pink, the second drug-free control well received the indicator and the above steps were repeated. Each minimum inhibitory concentration (MIC) was read on the 3^rd^ to 6^th^ days. The MIC was defined as the lowest drug concentration that prevented a change in color. The final result of MIC of each drug for each strain was the mean value from two tests. The MIC breakpoints of the drugs indicating sensitivity, moderate susceptibility, and resistance were interpreted according to the approved guidelines established by the National Committee for Clinical Laboratory Standards [4] and World Health Organization guidance [11] (Table 2) except that minocycle was according to Vanitha et al [12].

**Table 2:** The MIC breakpoints (µg/mL) of the 15 antimicrobial agents.

Antimicrobial agents	MIC breakpoints			References
	Sensitive	Moderate	Resistant	
Rifampicin	–	–	≥1	[4]
Isoniazid	–	–	≥1	[4]
Streptomycin	–	–	≥5	[4]
Amikacin	≤16	32	≥64	[4]
Kanamycin	–	–	≥4.0	[11]
Ciprofloxacin	≤1	2	≥4	[4]
Ofloxacin	–	–	≥2	[11]
Levofloxacin	≤2	4	≥8	[4]
Capreomycin	–	–	≥2.5	[11]
Cefoxitin	≤16	32–64	≥128	[4]
Doxycycline	≤1	2–8	≥16	[4]
Minocycline	8	16	≥32	[12]
Ethionamide	–	–	≥5.0	[11]
P-aminosalicylic acid	–	–	≥2.0	[11]
Dipasic	–	–	–	–

MIC, minimum inhibitory concentration.

## Results

The results of the antimicrobial susceptibility testing of the 12 standard RGM and 12 standard SGM strains are shown in Table 1. All 24 strains were highly resistant to isoniazid (all MIC > 256 µg/mL). Nine and six of 12 RGM strains were resistant to rifampicin and streptomycin, respectively, especially *Mycobacterium abscessus* and *Mycobacterium bolletii*, which showed very high resistance, while seven and two of 12 SGM strains were resistant to rifampicin and streptomycin, respectively. Among members of the *Mycobacterium chelonae-abscessus* complex, *M. abscessus* was more resistant to rifampicin than *M. chelonae*.

All of the NTM strains were sensitive or moderately susceptible to amikacin. However, most of the standard NTM strains (19/23) were resistant to the aminoglycoside kanamycin. The 12 SGM strains were all susceptible to amikacin, whereas only one SGM (*Mycobacterium senegalense*) was susceptible to kanamycin.

The fluoroquinolones were active against most of the NTM. Nine RGM strains (*Mycobacterium doricum*, *Mycobacterium fortuitum*, *Mycobacterium peregrinum*, *Mycobacterium obuense*, *Mycobacterium phlei*, *Mycobacterium duvalii*, *Mycobacterium parafortuitum*, *Mycobacterium gilvum*, and *Mycobacterium flavescens*) and 5 SGM strains (*Mycobacterium xenopi*, *Mycobacterium kansasii*, *Mycobacterium scrofulaceum*, *Mycobacterium malmoense*, and *Mycobacterium terrae*) were all susceptible to ciprofloxacin, ofloxacin, and levofloxacin.

All of the standard NTM strains were highly resistant to capreomycin (MIC > 256 µg/mL) except for *M. scrofulaceum* and *Mycobacterium gordonae*, which were low-level resistant (MIC = 4 µg/mL).

Twelve NTM strains (6/12 RGM strains and 6/12 SGM strains) were sensitive or moderately susceptible to the β-lactam antibiotic cefoxitin, while the other strains were highly resistant. Cefoxitin was the only antibiotic agent that *M. chelonae* was more resistant to than *M. abscessus* (>256 µg/mL vs. 64 µg/mL). Seven of 12 RGM strains (except *M. abscessus*, *M. chelonae*, *M. doricum*, *M. fortuitum*, *M. bolletii*) and 11 of 12 SGM (except *M. simiae*) strains were susceptible to minocycline.

Five RGM strains (*M. phlei*, *M. duvalii*, *M. parafortuitum*, *M. gilvum*, and *M. flavescens*) and ten SGM strains except *M. simiae* and *Mycobacterium. avium* were susceptible or moderately susceptible to doxycycline. Seven RGM strains (*M. peregrinum*, *M. fortuitum*, *M. phlei*, *M. duvalii*, *M. parafortuitum*, *M. gilvum*, and *M. flavescens*) and 11 SGM strains (all except *M. simiae*) were susceptible to minocycline.

Ethionamide is the derivative of isoniazid. Unlike the fact that all NTM strains were highly resistant to isoniazid, four of 12 RGM strains (*M. peregrinum, M. phlei*, *M. duvalii*, *M. gilvum*) and 6 of 12 SGM strains (*M. gordonae*, *Mycobacterium. marinum M. kansasii*, *M. scrofulaceum*, *M. malmoense*, and *M. avium*) were susceptible to ethionamide.

Dipasic is a mixture of p-aminosalicylic acid and isoniazid, and its MIC breakpoints (µg/mL) corresponded to those of p-aminosalicylic acid in this study (the MIC of dipasic for these 24 strains were all > 256 µg/mL). As such, all 24 NTM strains were resistant to p-aminosalicylic acid and dipasic.

## Discussion

Analyzing the susceptibility results of 24 standard NTM strains using the microplate Alamar Blue assay, it became evident that these findings yielded important clues for the optimization of NTM species-specific therapy. The results showed that streptomycin, amikacin, the fluoroquinolones, and the tetracyclines were the most active antimicrobial agents against the 12 RGM and 12 SGM strains. This is the first report of susceptibility patterns of standard NTM strains.

It is well know that most NTM strains are resistant to conventional anti-tuberculous agents[13]–[16], a fact that was further proven by the current findings. However, more SGM strains were susceptible to the drugs than RGM stains. Most of the SGM strains(10/12) were also susceptible to streptomycin. Several reports have shown that amikacin has good activity against RGM[7], [17]–[18]. In this study, 22 NTM strains were susceptible to and 2 NTM strains (*M. abscessus* and *M. chelonae*) were moderately susceptible to amikacin.

*M. abscessus* is naturally sensitive to amikacin, cefoxitin, and imipenem [19] and very resistant to many other chemotherapeutic agents [20]. In our study, *M. abscessus* was resistant to 13 antibiotics and moderately susceptible to amikacin and cefoxitin. These findings are comparable to those described in the other studies. A total of 38 (95%) isolates in a Taiwanese study (40 isolates of *M. abscessus* isolates obtained from January 2006 to December 2008) and 73 (99%) isolates in a Korean study (74 isolates of *M. abscessus* isolates obtained from July 2005 to December 2006) of *in vitro* antimicrobial susceptibility were sensitive to amikacin[21]–[22]. *M. abscessus* and *M. chelonae* are members of the *M. chelonae-abscessus* complex, the susceptibility patterns of which are similar, although *M. abscessus* was more resistant than *M. chelonae* to rifampicin, ciprofloxacin, ofloxacin, and levofloxacin. Cefoxitin was the only antibiotic agent tested here that *M. chelona*e was more resistant to than *M. abscessus* (>256 µg/mL vs. 64 µg/mL), suggesting that cefoxitin resistance could be a way to distinguish between *M. chelona*e and *M. abscessus*.

*M. fortuitum* and *M. peregrinum* are members of the *M. fortuitum* complex. However, *M. peregrinum* was more sensitive to rifampicin, streptomycin, amikacin, cefoxitin, doxycycline, minocycline, and ethionamide than was *M. fortuitum*. Studies have reported that the *M. fortuitum* complex was much less drug-resistant than *M. abscessus* and *M. chelonae* [16], [23]–[25]; in our study, the *M. fortuitum* complex was more susceptible to amikacin, kanamycin, ciprofloxacin, ofloxacin, and levofloxacin than *M. abscessus* and *M. chelonae.*

*M. bolletii* and *M. simiae* were resistant to 14 of 15 antibiotic agents and susceptible to amikacin, so more agents should be included in future tests. One study showed that 38% (11/29), 25% (7/29), 100% (29/29), 90% (26/29), and 66% (19/29)of *M. simiae* clinical isolates were susceptible to ciprofloxacin, clarithromycin, cycloserine, clofazimine, and prothionamide, respectively [21]. However, the efficacy of these drugs in NTM treatment has not been sufficiently proven and they are limited by their toxicity [13], [26].

**Figure 1:**
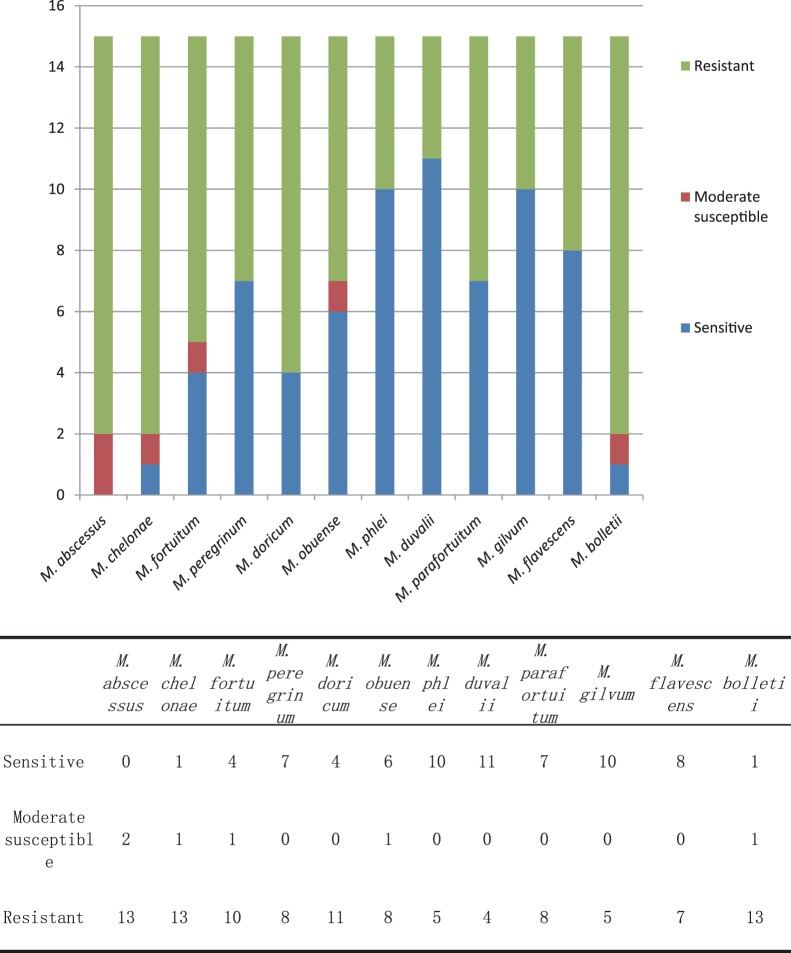
The susceptibility distributions to 15 antimicrobial agents of 12 standard rapidly growing mycobacteria strains.

**Figure 2:**
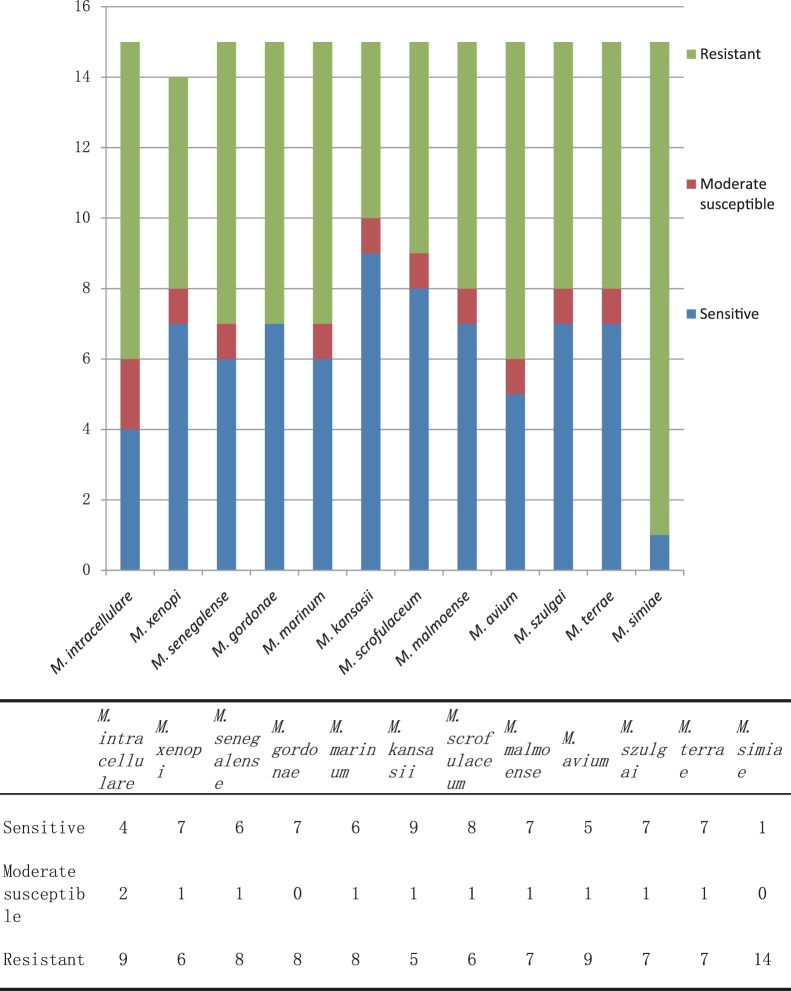
The susceptibility distributions to 15 antimicrobial agents of 12 standard slowly growing mycobacteria strains.

In summary, *M. abscessus*, *M. chelonae*, and *M. bolletii* were resistant to almost all 15 antimicrobial agents, while the other nine standard RGM strains were resistant to 4–11 drugs (Figure 1). A total of 11 SGM standard strains were resistant to 6–11 drugs, while *M. simiae* was resistant to 14 drugs (Figure 2).

The American Thoracic Society advocated the use of macrolide-based multidrug regimens for NTM treatment [13], and some studies have reported that most NTM strains are sensitive to the macrolide clarithromycin [6], [27] and that rifabutin and tigecycline also showed high activity against many NTM strains [6], [17]. As such, streptomycin, amikacin, the fluoroquinones, the tetracyclines, and the above three antibiotics were the alternative choices for the treatment of NTM infection. Studies have shown that the susceptibilities of clinical NTM isolates of a species were also quite different [21], [27], so our results from NTM standard strains can only be referenced by clinicians before susceptibility testing for clinical isolates is performed or when conditions do not allow for susceptibility testing. Susceptibility testing for clinical isolates should always be performed prior to treatment unless conditions do not permit such.

The technique described here can offer the MIC of antimicrobial agents within 6 days. The microplate Alamar Blue assay is inexpensive and reliable for the DST of NTM. Alternatively, the application of broth-based methods is recommended by the CLSI and the susceptibility patterns of standard strains of *Mycobacterium* can improve the international standardization of susceptibility testing methods.

## References

[1] World health organization. Global tuberculosis control: WHO report 2011.

[2] Richter E, Rusch-Gerdes S, Hillemann D (2006) Evaluation of the GenoType Mycobacterium Assay for identification of mycobacterial species from cultures. J Clin Microbiol 44: 1769–1775. 16672405 10.1128/JCM.44.5.1769-1775.2006 PMC1479216

[3] Bicmen C, Gunduz AT, Coskun M, Senol G, Cirak AK, et al (2011) Molecular detection and identification of *mycobacterium tuberculosis* complex and four clinically important nontuberculous mycobacterial species in smear-negative clinical samples by the genotype mycobacteria direct test. J Clin Microbiol 49: 2874–2878. 21653780 10.1128/JCM.00612-11 PMC3147717

[4] National Committee for Clinical Laboratory Standards. Susceptibility testing of *mycobacteria*, *Nocardiae* and other *aerobic actinomycetes*; approved standard. Document M24-A.Wayne, PA: NCCLS; 2003. 31339680

[5] Garcia-Agudo L, Garcia-Martos P, Jesus I, Rodriguez-Iglesias M (2009) Assessment of in vitro susceptibility to antimicrobials of rapidly growing mycobacteria by E-test. Rev Med Chil 137: 912–917. 19802419

[6] van Ingen J, van der Laan T, Dekhuijzen R, Boeree M, van Soolingen D (2010) In vitro drug susceptibility of 2275 clinical non-tuberculous Mycobacterium isolates of 49 species in The Netherlands. Int J Antimicrob Agents 35: 169–173. 20006470 10.1016/j.ijantimicag.2009.09.023

[7] Gayathri R, Therese KL, Deepa P, Mangai S, Madhavan HN (2010) Antibiotic susceptibility pattern of rapidly growing mycobacteria. J Postgrad Med 56: 76–78. 20622384 10.4103/0022-3859.65278

[8] Aubry A, Jarlier V, Escolano S, Truffot-Pernot C, Cambau E (2000) Antibiotic susceptibility pattern of *Mycobacterium marinum* . Antimicrob Agents Chemother 44: 3133–3136. 11036036 10.1128/aac.44.11.3133-3136.2000 PMC101616

[9] Leonard B, Coronel J, Siedner M, Grandjean L, Caviedes L, et al (2008) Inter- and intra-assay reproducibility of microplate Alamar blue assay results for isoniazid, rifampicin, ethambutol, streptomycin, ciprofloxacin, and capreomycin drug susceptibility testing of *Mycobacterium tuberculosis* . J Clin Microbiol 46: 3526–3529. 18701659 10.1128/JCM.02083-07 PMC2566109

[10] Chauca JA, Palomino JC, Guerra H (2007) Evaluation of the accuracy of the microplate Alamar Blue assay for rapid detection of MDR-TB in Peru. Int J Tuberc Lung Dis 11: 820–822. 17609061

[11] Policy guidance on drug-susceptibility testing (DST) of second-line antituberculosis drugs. World Health Organization, Geneva, 2008. 26290924

[12] Vanitha JD, Paramasivan CN (2004) Evaluation of microplate Alamar blue assay for drug susceptibility testing of *Mycobacterium avium* complex isolates. Diagn Microbiol Infect Dis 49: 179–182. 15246507 10.1016/j.diagmicrobio.2004.04.003

[13] Griffith DE, Aksamit T, Brown-Elliott BA, Catanzaro A, Daley C, et al (2007) An official ATS/IDSA statement: diagnosis, treatment, and prevention of nontuberculous mycobacterial diseases. Am J Respir Crit Care Med 175: 367–416. 17277290 10.1164/rccm.200604-571ST

[14] Yang SC, Hsueh PR, Lai HC, Teng LJ, Huang LM, et al (2003) High prevalence of antimicrobial resistance in rapidly growing mycobacteria in Taiwan. Antimicrob Agents Chemother 47: 1958–1962. 12760874 10.1128/AAC.47.6.1958-1962.2003 PMC155839

[15] Bicmen C, Coskun M, Gunduz AT, Senol G, Cirak AK, et al (2010) Nontuberculous mycobacteria isolated from pulmonary specimens between 2004 and 2009: causative agent or not? New Microbiol 33: 399–403. 21213600

[16] Brown-Elliott BA, Wallace RJ Jr (2002) Clinical and taxonomic status of pathogenic nonpigmented or late-pigmenting rapidly growing mycobacteria. Clin Microbiol Rev 15: 716–746. 12364376 10.1128/CMR.15.4.716-746.2002 PMC126856

[17] Fernandez-Roblas R, Martin-de-Hijas NZ, Fernandez-Martinez AI, Garcia-Almeida D, Gadea I, et al (2008) In vitro activities of tigecycline and 10 other antimicrobials against nonpigmented rapidly growing mycobacteria. Antimicrob Agents Chemother 52: 4184–4186. 18725441 10.1128/AAC.00695-08 PMC2573152

[18] Shen GH, Wu BD, Wu KM, Chen JH (2007) In Vitro activities of isepamicin, other aminoglycosides, and capreomycin against clinical isolates of rapidly growing mycobacteria in Taiwan. Antimicrob Agents Chemother 51: 1849–1851. 17353252 10.1128/AAC.01551-06 PMC1855558

[19] Petrini B (2006) *Mycobacterium abscessus*: an emerging rapid-growing potential pathogen. APMIS 114: 319–328. 16725007 10.1111/j.1600-0463.2006.apm_390.x

[20] Colombo RE, Olivier KN (2008) Diagnosis and treatment of infections caused by rapidly growing mycobacteria. Semin Respir Crit Care Med 29: 577–588. 18810691 10.1055/s-0028-1085709

[21] Huang YC, Liu MF, Shen GH, Lin CF, Kao CC, et al (2010) Clinical outcome of *Mycobacterium abscessus* infection and antimicrobial susceptibility testing. J Microbiol Immunol Infect 43: 401–406. 21075707 10.1016/S1684-1182(10)60063-1

[22] Park S, Kim S, Park EM, Kim H, Kwon OJ, et al (2008) In vitro antimicrobial susceptibility of *Mycobacterium abscessus* in Korea. J Korean Med Sci 23: 49–52. 18303198 10.3346/jkms.2008.23.1.49 PMC2526484

[23] Wallace RJ Jr, Brown-Elliott BA, Crist CJ, Mann L, Wilson RW (2002) Comparison of the in vitro activity of the glycylcycline tigecycline (formerly GAR-936) with those of tetracycline, minocycline, and doxycycline against isolates of nontuberculous mycobacteria. Antimicrob Agents Chemother 46: 3164–3167. 12234839 10.1128/AAC.46.10.3164-3167.2002 PMC128779

[24] Wallace RJ Jr, Bedsole G, Sumter G, Sanders CV, Steele LC, et al (1990) Activities of ciprofloxacin and ofloxacin against rapidly growing mycobacteria with demonstration of acquired resistance following single-drug therapy. Antimicrob Agents Chemother 34: 65–70. 2327761 10.1128/aac.34.1.65 PMC171521

[25] Wallace RJ Jr, Brown BA, Onyi GO (1991) Susceptibilities of *Mycobacterium fortuitum biovar. fortuitum* and the two subgroups of *Mycobacterium chelonae* to imipenem, cefmetazole, cefoxitin, and amoxicillin-clavulanic acid. Antimicrob Agents Chemother 35: 773–775. 2069387 10.1128/aac.35.4.773 PMC245098

[26] David S (2001) Synergic activity of D-cycloserine and beta-chloro-D-alanine against *Mycobacterium tuberculosis* . J Antimicrob Chemother 47: 203–206. 11157908 10.1093/jac/47.2.203

[27] Cavusoglu C, Gurpinar T, Ecemis T (2012) Evaluation of antimicrobial susceptibilities of rapidly growing mycobacteria by Sensititre RAPMYCO panel. New Microbiol 35: 73–76. 22378556

